# Ritlecitinib, a JAK3 /TEC inhibitor, modulates the markers of celiac autoimmunity in alopecia areata and vitiligo patients

**DOI:** 10.1007/s00403-024-03784-6

**Published:** 2025-01-18

**Authors:** Rok Seon Choung, Jyoti Ramakrishna, Vivek Pradhan, Brett King, Emma Guttman-Yassky, Elena Peeva, Joseph A. Murray

**Affiliations:** 1 Division of Gastroenterology and Hepatology, Mayo Clinic, Rochester, MN USA; 2 Inflammation and Immunology Research Unit, Pfizer R&D, Cambridge, MA USA; 3 Department of Dermatology, Yale School of Medicine, New Haven, Connecticut, USA; 4 Department of Dermatology and the Immunology Institute, Icahn School of Medicine at Mount Sinai, New York, NY USA; 5 Division of Gastroenterology and Hepatology, 200 1st Street SW, Rochester, MN 55905 USA

**Keywords:** Ritlecitinib, Celiac autoimmunity, Alopecia areata, Vitiligo

## Abstract

**Background:**

Celiac disease (CeD) has shown an association with autoimmune disorders including vitiligo and alopecia areata (AA). Ritlecitinib, a JAK3 and TEC kinase family inhibitor, has been approved for treatment of patients with AA and is in late-stage development for vitiligo. Ritlecitinib inhibits cytotoxic T cells, NK cells, and B cells which play a role in the pathogenesis of CeD.

**Objective:**

We aimed to explore the potential effect of ritlecitinib on CeD serology levels before and after ritlecitinib treatment in research participants of clinical trials.

**Methods:**

The effect of ritlecitinib on CeD serology (tTG-IgA, DGP-IgA/IgG) levels was retrospectively evaluated in participants from three phase 2 and one phase 3 ritlecitinib clinical trials including participants with active AA, rheumatoid arthritis (RA) and vitiligo, whose serum samples at baseline and post-treatment were available. All statistical comparisons of the changes between initial and follow-up samples used the Wilcoxon matched pairs exact test.

**Results:**

Of 1146 research participants, 21 individuals had a positive tTG-IgA in their baseline samples (positivity rate, 0.018, 95% CI = 0.011–0.028). Among these 21 individuals, follow-up samples were available in 15 participants from the ritlecitinib group and in 3 from the placebo group. In follow-up samples, the values of tTG-IgA in the 15 participants treated with ritlecitinib significantly decreased from baseline (*p* < 0.01), while in the placebo group the tTGA-IgA levels remained close to the baseline values.

**Conclusion:**

A decrease in CeD serology levels with ritlecitinib treatment suggests that ritlecitinib may provide beneficial effect in CeD.

## Introduction

Celiac disease (CeD) is a relatively common autoimmune disease that causes malabsorption due to gluten-induced inflammatory mucosal damage in the small intestine [1]. The association of CeD with other autoimmune diseases including alopecia areata, vitiligo and rheumatoid arthritis has been reported [2, 3].

There are no approved treatments for CeD. A gluten-free diet (GFD) is the only therapy for CeD, and more than 30% of celiac patients have persistent symptoms and abnormal serologies despite following GFD [1, 4]. Thus, there is significant unmet need for effective treatment options for CeD. Ritlecitinib, which inhibits Janus kinase (JAK) 3 and all five members of the tyrosine kinase expressed in hepatocellular carcinoma (TEC) kinase family, is approved for treatment of severe AA and is in clinical trials for vitiligo [5, 6]. Previously, ritlecitinib was also evaluated in RA patients. Exploring the hypothesis that ritlecitinib may improve CeD, we identified patients with positive CeD serologies in three cohorts from four completed ritlecitinib clinical trials in AA, vitiligo and RA, and compared CeD serologies at baseline and after treatment with ritlecitinib or placebo.

## Results and discussion

Blood samples from 1,146 research participants in four randomized placebo-controlled clinical trials of ritlecitinib for alopecia areata (AA), vitiligo, or rheumatoid arthritis (RA) were analyzed for celiac disease (CeD) serologies. This included testing for tissue transglutaminase IgA, deamidated gliadin IgA and IgG, and IgA endomysium antibodies.

At baseline, 21 study subjects out of the 1146 subjects had a positive tTG-IgA (positivity rate of 0.018 (95% CI = 0.011–0.028). Of these 21 individuals with positive tTG-IgA, 9 were positive for all CeD serologies and 14 showed positivity for at least one additional CeD serological test (DGP-IgA, DGP-IgG, or EMA). Demographics and baseline characteristics of tTG-IgA-positive individuals were similar across the treatment groups (Table 1). Approximately 1.8% of patients with vitiligo, AA and RA who were enrolled in the clinical trials with ritlecitinib had positive tTG-IgA. This observation is similar to the CeD serology prevalence reported in previous studies, ranging from 0.9 to 2.9% [2, 7].

**Table 1:** Demographics and baseline characteristics of individuals with positive tTG-IgA according to the treatment groups

	Ritlecitinib (*N* = 15)	Placebo (*N* = 3)	Total (*N* = 18)
Age (yrs)	37.4 ± 15.59 [12,42,66](15)	37.7 ± 17.90 [18,42,53](3)	37.4 ± 15.42 [12,42,66](18)
Gender: Male	46.7% (7/15)	0.0% (0/3)	38.9% (7/18)
Ethnicity			
White	80.0% (12/15)	66.7% (2/3)	77.8% (14/18)
Asian	20.0% (3/15)	33.3% (1/3)	22.2% (4/18)
**Autoimmune disease**			
Alopecia Areata	66.7% (10/15)	66.7% (2/3)	66.7% (12/18)
Vitiligo	26.7% (4/15)	33.3% (1/3)	27.8% (5/18)
RA	6.7% (1/15)	0.0% (0/3)	5.6% (1/18)
**Duration of treatment at final sample collection (weeks)**	13.7 ± 11.66 [4,12,48](15)	16.0 ± 6.69 [12,12,24](3)	14.1 ± 10.86 [4,12,48](18)

Continuous measures are Mean ± Std [Min, Median, Max] (N)

Of the 21 individuals with positive tTG -IgA levels at baseline, 17 had 24 additional serum samples collected during the course of the trials. One patient received both placebo followed by ritlecitinib, and had paired samples from both treatment periods. CeD serologies including tTG-IgA, DGP-IgA, and DGP-IgG were significantly reduced after treatment with ritlecitinib while in the placebo group, CeD serology levels remained similar to the baseline values (Table 2).

**Table 2:** Celiac disease serology results before and after treatment with ritlecitinib or placebo

	Ritlecitinib (*N* = 15)	Placebo (*N* = 3)
**tTG-IgA**		
Baseline	31.8 ± 35.3 [4.1,17.1,100.0](15)	22.9 ± 20.5 [9.3,12.9,46.5](3)
Follow-up	14.8 ± 18.6 [1.8,7.4,73.9](15)	21.3 ± 14.6 [5.6,23.8,34.5](3)
p-value*	0.003	0.750
**DGP-IgA**		
Baseline	47.3 ± 65.2 [2.2,19.2, 216.4] (15)	107.7 ± 102.8 [14.2, 91.1, 217.8](3)
Follow-up	20.4 ± 26.7 [2.6,12.5,100.8](15)	112.6 ± 99.1 [10.2,119.4,208.1](3)
p-value*	0.018	1.000
**DGP-IgG**		
Baseline	36.1 ± 41.2 [0.3,27.0,132.7](15)	47.9 ± 8.9 [40.2,45.8,57.7](3)
Follow-up	19.4 ± 21.3 [0.3,10.9,66.8](15)	45.7 ± 13.8 [30.1,50.9,56.2](3)
p-value*	0.002	1.000

Continuous measures are Mean ± Std [Min, Median, Max] (N)

*p-value calculated using 2-sided exact Wilcoxon-signed rank test

Currently, there are no approved medications for CeD. Avoiding exposure to gluten is the only available option, but this can be very challenging and CeD patients struggle constantly to adhere to GFD, often facing inadvertent exposures to gluten with undesirable consequences. Moreover, more than 30% of CeD patients are non-responsive to GFD in spite of strict adherence to GFD [1, 4], highlighting the importance of therapeutic development in CeD [8].

Comparing CeD serologies (tTG-IgA, DGP-IgA, and DGP-IgG) before and after treatment with ritlecitinib, we found significant reduction in values of CeD antibody levels in patients who were positive for tTG-IgA at baseline. By inhibiting JAK3 and TEC kinases, ritlecitinib modulates CD8 + T cell and NK cell function by inhibition of IL15, IFN gamma and associated chemokines (e.g. CXCL10, CXCL9 and others), and also downregulates B cell functions, including autoantibody secretion, by inhibition of Type 2 cytokines such as IL-13, IL-9, and CCL18, with correlations between biomarker changes and clinical responses [9].

CeD may be diagnosed based on CeD serologies, especially in pediatric cases [10, 11]. Pediatric guidelines support high levels of tTG-IgA as a diagnostic marker, eliminating the need for endoscopic biopsies [11, 12]. tTG-IgA levels typically decrease and normalize after starting a GFD, although this may take over 6 months [13, 14]. Therefore, the rapid reduction in tTG-IgA levels with ritlecitinib is quite impressive and indicates that ritlecitinb may provide beneficial effect in CeD.

The current study has several limitations, including the post-hoc analysis of blood samples from patients with autoimmune diseases participating in ritlecitinib clinical trials with heterogeneous dosing regimens across a small number of patients. The very small placebo group prevented direct comparison between the placebo and ritlecitinib groups. In particular, although the baseline CeD serology values seem numerically different between the two groups, they are not statistically different (the p-values are > 0.05 for all three serological parameters). Also, given that there are only three patients in the placebo group, it is not reliable to draw any conclusions. It is unknown if the patients in the current study had clinical CeD. As the patients were in unrelated clinical trials, diet histories, which could affect the tTG-IgA values, were not taken. However, the rapid decrease in tTG-IgA levels (median time of 84 days) indicated an impressive effect of ritlecitinib on CeD serologies. As already mentioned, usually takes over six months for tTG-IgA levels to decrease and normalize after starting a GFD [13, 14], not to mention that many celiac patients do not normalize tTG-IgA values even after six months of a GFD [15].

In conclusion, the current analysis confirms that a subset of patients with autoimmune diseases have positive CeD serology. Rapid reduction of CeD serologies in ritlecitinib-treated patients suggests a potential for therapeutic effect in CeD and merits further evaluation of ritlecitinib for treatment of CeD.

## Data Availability

No datasets were generated or analysed during the current study.
