# Correction: Ritlecitinib, a JAK3 /TEC inhibitor, modulates the markers of celiac autoimmunity in alopecia areata and vitiligo patients

**DOI:** 10.1007/s00403-025-04136-8

**Published:** 2025-03-12

**Authors:** Rok Seon Choung, Jyoti Ramakrishna, Vivek Pradhan, Brett King, Emma Guttman-Yassky, Elena Peeva, Joseph A. Murray

**Affiliations:** 1 Division of Gastroenterology and Hepatology, Mayo Clinic, Rochester, MN USA; 2 Inflammation and Immunology Research Unit, Pfizer R&D, Cambridge, MA USA; 3 Department of Dermatology, Yale School of Medicine, New Haven, Connecticut, USA; 4 Department of Dermatology and the Immunology Institute, Icahn School of Medicine at Mount Sinai, New York, NY USA; 5 Division of Gastroenterology and Hepatology, 200 1st Street SW, Rochester, MN 55905 USA

**Archives of Dermatological Research (2025) 317:280**

10.1007/s00403-024-03784-6

The article “Ritlecitinib, a JAK3 /TEC inhibitor, modulates the markers of celiac autoimmunity in alopecia areata and vitiligo patients”, written by Rok Seon Choung, Jyoti Ramakrishna, Vivek Pradhan, Brett King, Emma Guttman-Yassky, Elena Peeva and Joseph A. Murray, was originally published under exclusive license to Springer-Verlag GmbH Germany, part of Springer Nature. As a result of the subsequent decision to publish the article under the open access model, the article’s copyright notice was changed on 18 February 2025 to © The Author(s) 2025 and the article is now distributed under a Creative Commons Attribution 4.0 International License, which permits use, sharing, adaptation, distribution and reproduction in any medium or format, as long as you give appropriate credit to the original author(s) and the source, provide a link to the Creative Commons licence, and indicate if changes were made. The images or other third party material in this article are included in the article’s Creative Commons licence, unless indicated otherwise in a credit line to the material. If material is not included in the article’s Creative Commons licence and your intended use is not permitted by statutory regulation or exceeds the permitted use, you will need to obtain permission directly from the copyright holder.

To view a copy of this licence, visit http://creativecommons.org/licenses/by/4.0.

The original article has been corrected.

